# Supplementary material for: Alteration of the oral and gut microbiota in patients with Kawasaki disease
Source: PeerJ. 2023 Jul 10;11:e15662. doi: 10.7717/peerj.15662 (PMC10340105; doi:10.7717/peerj.15662)
Supplement: Supplemental Information 1 [file peerj-11-15662-s001.docx]

**Table S1 The basic information of all samples**

| **Sample\Info** | **KD or healthy** | **Age (months)** | **Gender** | **Fever** | **Pre-existing diseases** | **Diet** |
| --- | --- | --- | --- | --- | --- | --- |
| F1 | KD | 36 M | Male | Yes | No | Breastfeeding stop at 26 M^*^ |
| F2 | KD | 28 M | Female | Yes | Pneumonia | Bottle-feeding |
| F3 | KD | 29 M | Female | Yes | No | Breastfeeding, |
| F4 | KD | 21 M | Male | Yes | Pneumonia | Breastfeeding |
| F5 | KD | 28 M | Female | Yes | No | Bottle-feeding |
| F7 | Healthy | 39 M | Male | Yes | No | Breastfeeding stop at 24 M |
| F8 | Healthy | 23 M | Male | Yes | No | Breastfeeding |
| F9 | Healthy | 31 M | Female | Yes | No | Breastfeeding |
| F10 | Healthy | 38 M | Female | Yes | No | Breastfeeding stop at 30 M |
| O1 | KD | 36 M | Male | Yes | No | Breastfeeding stop at 26 M |
| O2 | KD | 28 M | Female | Yes | Pneumonia | Bottle-feeding |
| O3 | KD | 29 M | Female | Yes | No | Breastfeeding, |
| O4 | KD | 21 M | Male | Yes | Pneumonia | Breastfeeding |
| O5 | KD | 28 M | Female | Yes | No | Bottle-feeding |
| O7 | Healthy | 39 M | Male | Yes | No | Breastfeeding stop at 24 M |
| O8 | Healthy | 23 M | Male | Yes | No | Breastfeeding |
| O9 | Healthy | 31 M | Female | Yes | No | Breastfeeding |
| O10 | Healthy | 38 M | Female | Yes | No | Breastfeeding stop at 30 M |
| O11 | Healthy | 33 M | Male | Yes | No | Breastfeeding stop at 24 M |

^*:^ After breastfeeding, artificial feeding was commonly processed.
