# Supplementary material for: Alteration of the oral and gut microbiota in patients with Kawasaki disease
Source: PeerJ. 2023 Jul 10;11:e15662. doi: 10.7717/peerj.15662 (PMC10340105; doi:10.7717/peerj.15662)
Supplement: Supplemental Information 2 [file peerj-11-15662-s002.docx]

**Table S2 The sequences information of all samples**

| **Sample\Info** | **Seq_num** | **Base_num** | **Mean_length** | **Min_length** | **Max_length** | **Source** |
| --- | --- | --- | --- | --- | --- | --- |
| F1 | 38291 | 16102077 | 420.5186 | 256 | 432 | Patient |
| F2 | 40560 | 17368567 | 428.2191 | 403 | 431 | Patient |
| F3 | 41855 | 17837907 | 426.1834 | 255 | 463 | Patient |
| F4 | 36404 | 15584158 | 428.0892 | 403 | 430 | Patient |
| F5 | 52717 | 21530780 | 408.422 | 216 | 519 | Patient |
| F7 | 40650 | 16857522 | 414.6992 | 401 | 432 | Control |
| F8 | 52345 | 21666447 | 413.9163 | 400 | 478 | Control |
| F9 | 53597 | 22332087 | 416.6667 | 337 | 431 | Control |
| F10 | 42767 | 17751737 | 415.0802 | 337 | 430 | Control |
| O1 | 44072 | 18683304 | 423.9268 | 270 | 509 | Patient |
| O2 | 33756 | 14416047 | 427.0662 | 396 | 431 | Patient |
| O3 | 36866 | 15721653 | 426.454 | 266 | 431 | Patient |
| O4 | 35873 | 15376315 | 428.632 | 222 | 432 | Patient |
| O5 | 43969 | 18500360 | 420.7592 | 212 | 516 | Patient |
| O7 | 48742 | 20601022 | 422.6544 | 257 | 507 | Control |
| O8 | 58976 | 25011747 | 424.1004 | 212 | 510 | Control |
| O9 | 44865 | 18975497 | 422.9466 | 223 | 478 | Control |
| O10 | 37642 | 16006148 | 425.2204 | 261 | 507 | Control |
| O11 | 52839 | 22375571 | 423.467 | 220 | 527 | Control |
