# Supplementary figures and images for: Alteration of the oral and gut microbiota in patients with Kawasaki disease
Source: PeerJ. 2023 Jul 10;11:e15662. doi: 10.7717/peerj.15662 (PMC10340105; doi:10.7717/peerj.15662)

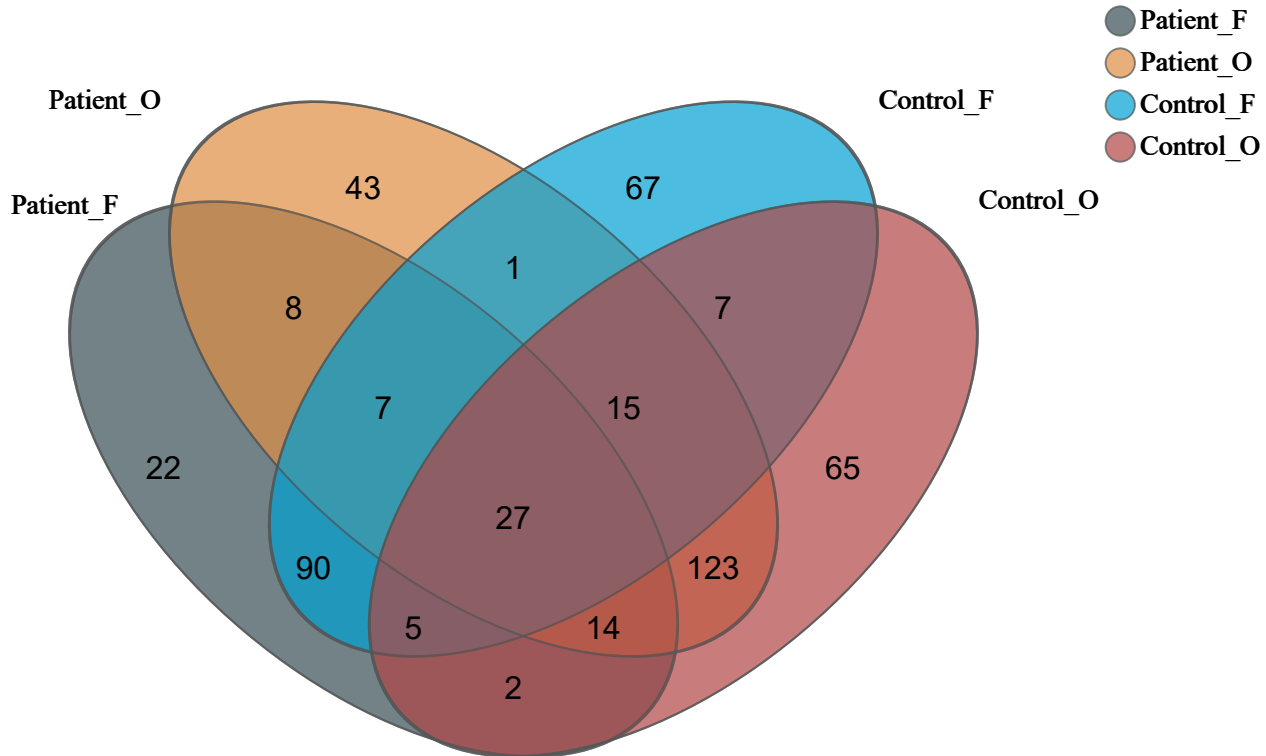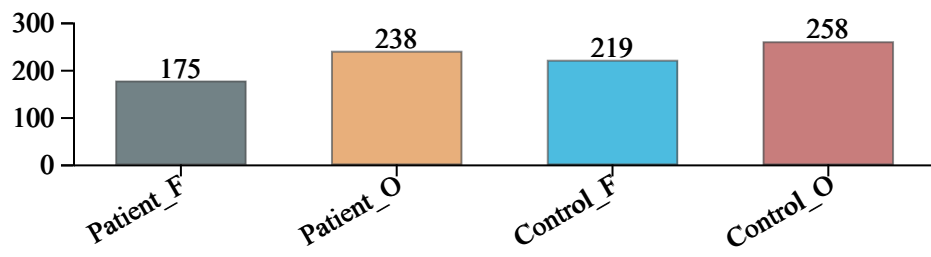

Supplement: Supplemental Information 3 — Patient F and Control F represent fecal samples from KD patients and health, respectively; Patient O and Control O represent oral samples from KD patients and health, respectively [file peerj-11-15662-s003.pdf]

Rarefaction curves

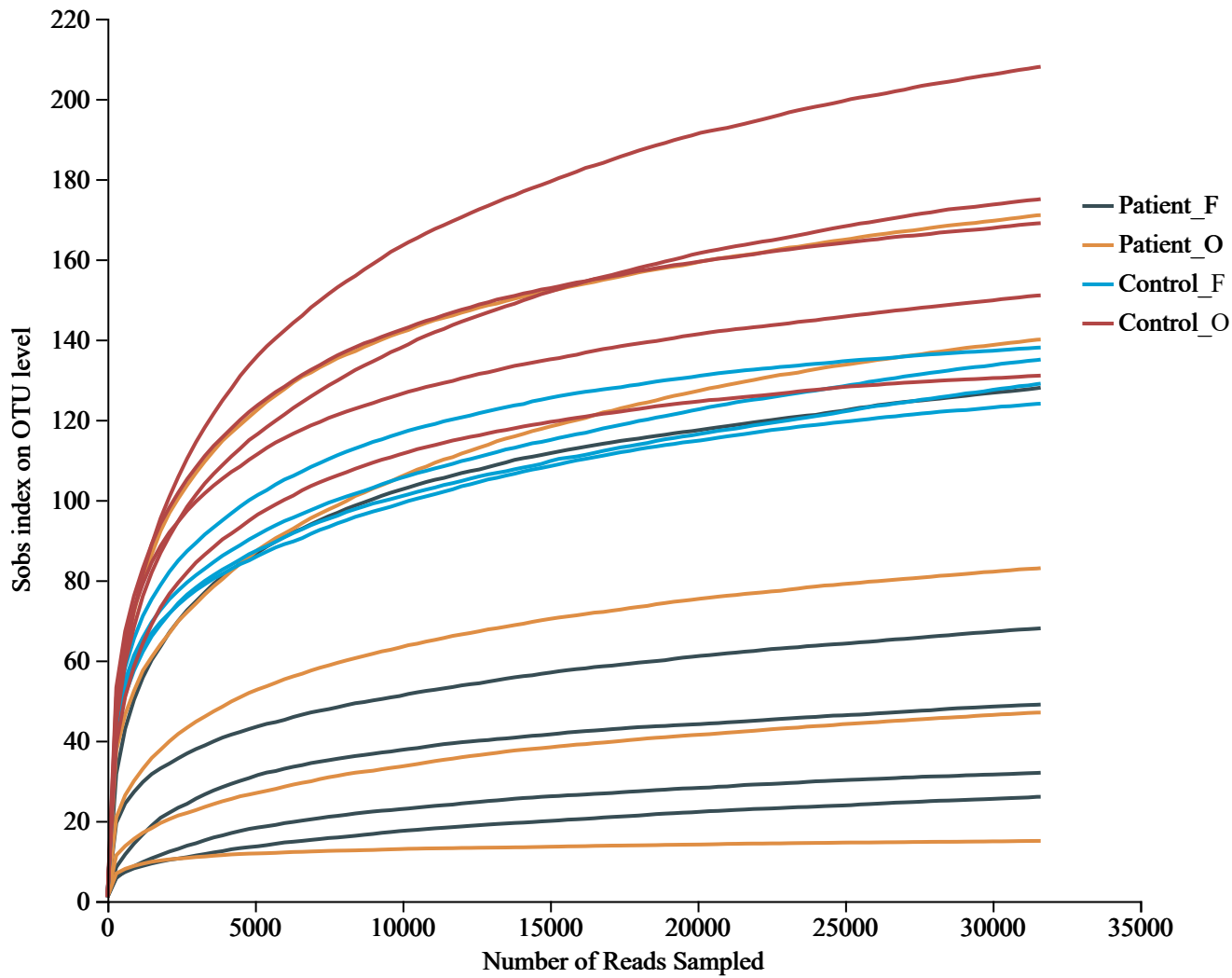

Supplement: Supplemental Information 4 — atient F and Control F represent fecal samples from KD patients and health, respectively; Patient O and Control O represent oral samples from KD patients and health, respectively [file peerj-11-15662-s004.pdf]

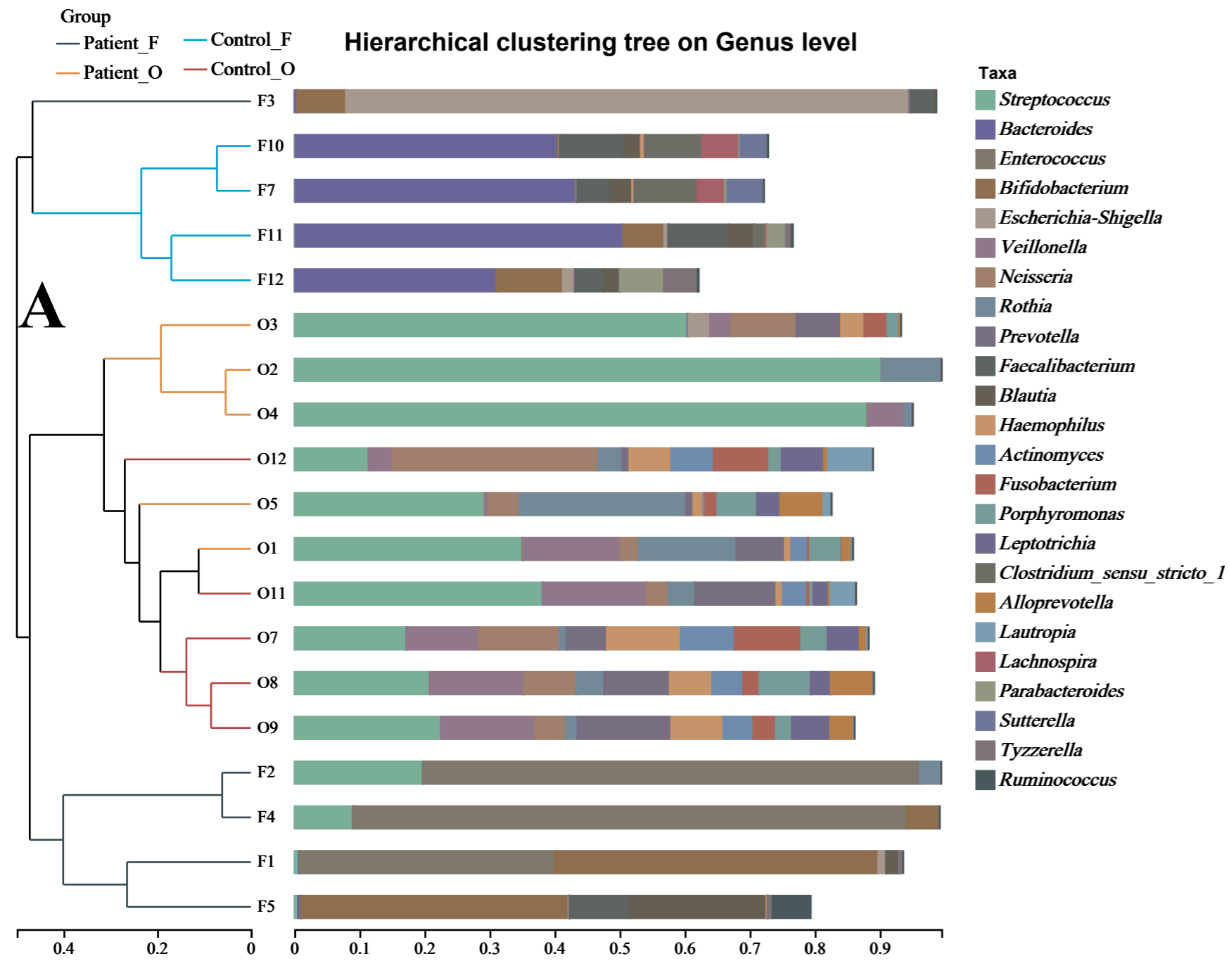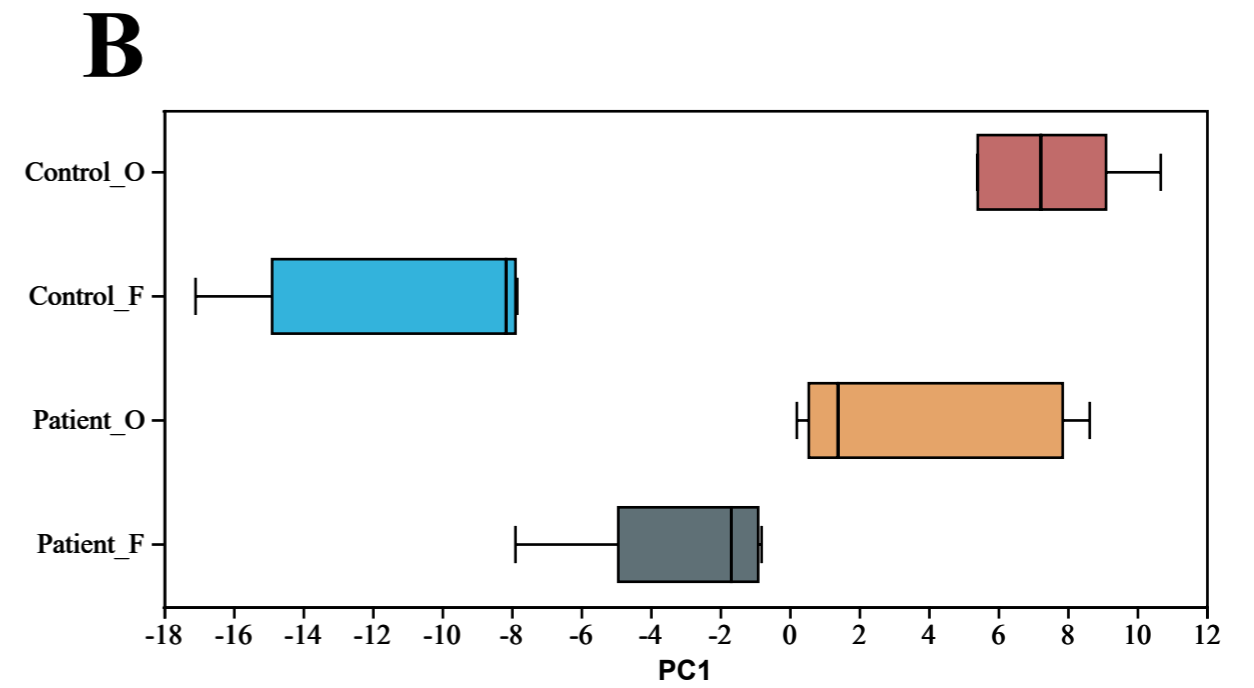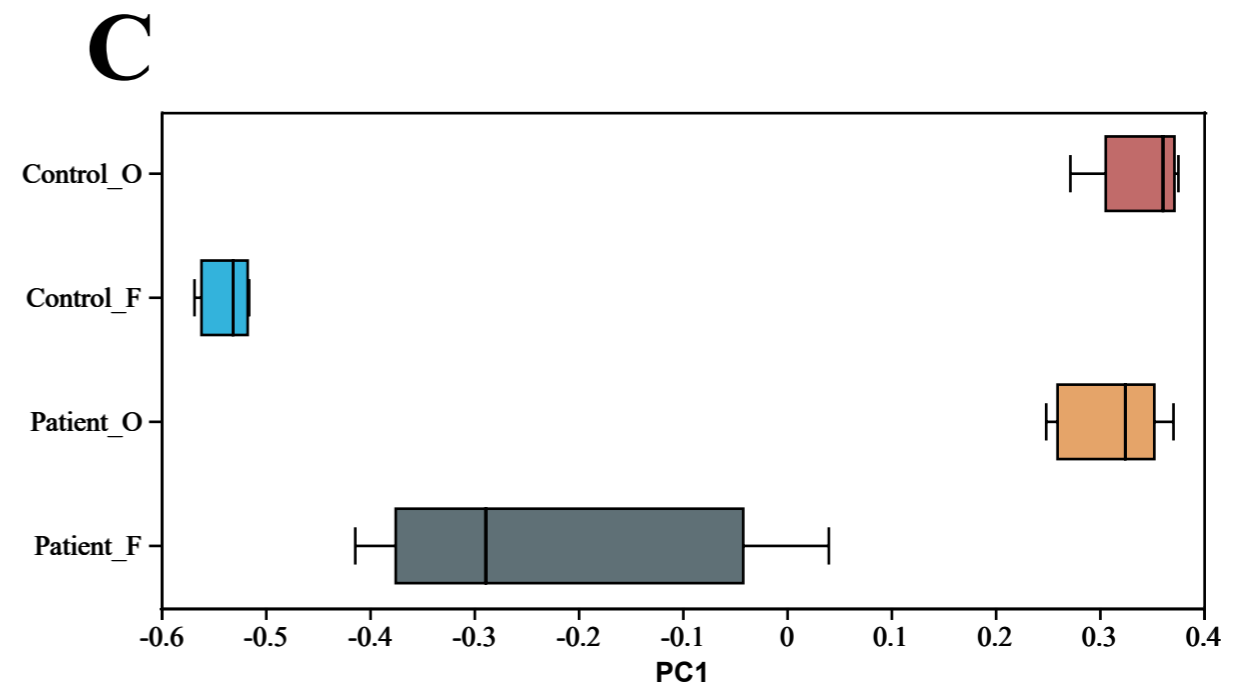

Supplement: Supplemental Information 5 — Patient F and Control F represent fecal samples from KD patients and health, respectively; Patient O and Control O represent oral samples from KD patients and health, respectively [file peerj-11-15662-s005.pdf]
