# Supplementary material for: Alteration of the oral and gut microbiota in patients with Kawasaki disease
Source: PeerJ. 2023 Jul 10;11:e15662. doi: 10.7717/peerj.15662 (PMC10340105; doi:10.7717/peerj.15662)
Supplement: Supplemental Information 6 — Patient F and Control F represent fecal samples from KD patients and health, respectively; Patient O and Control O represent oral samples from KD patients and health, respectively [file peerj-11-15662-s006.pdf]

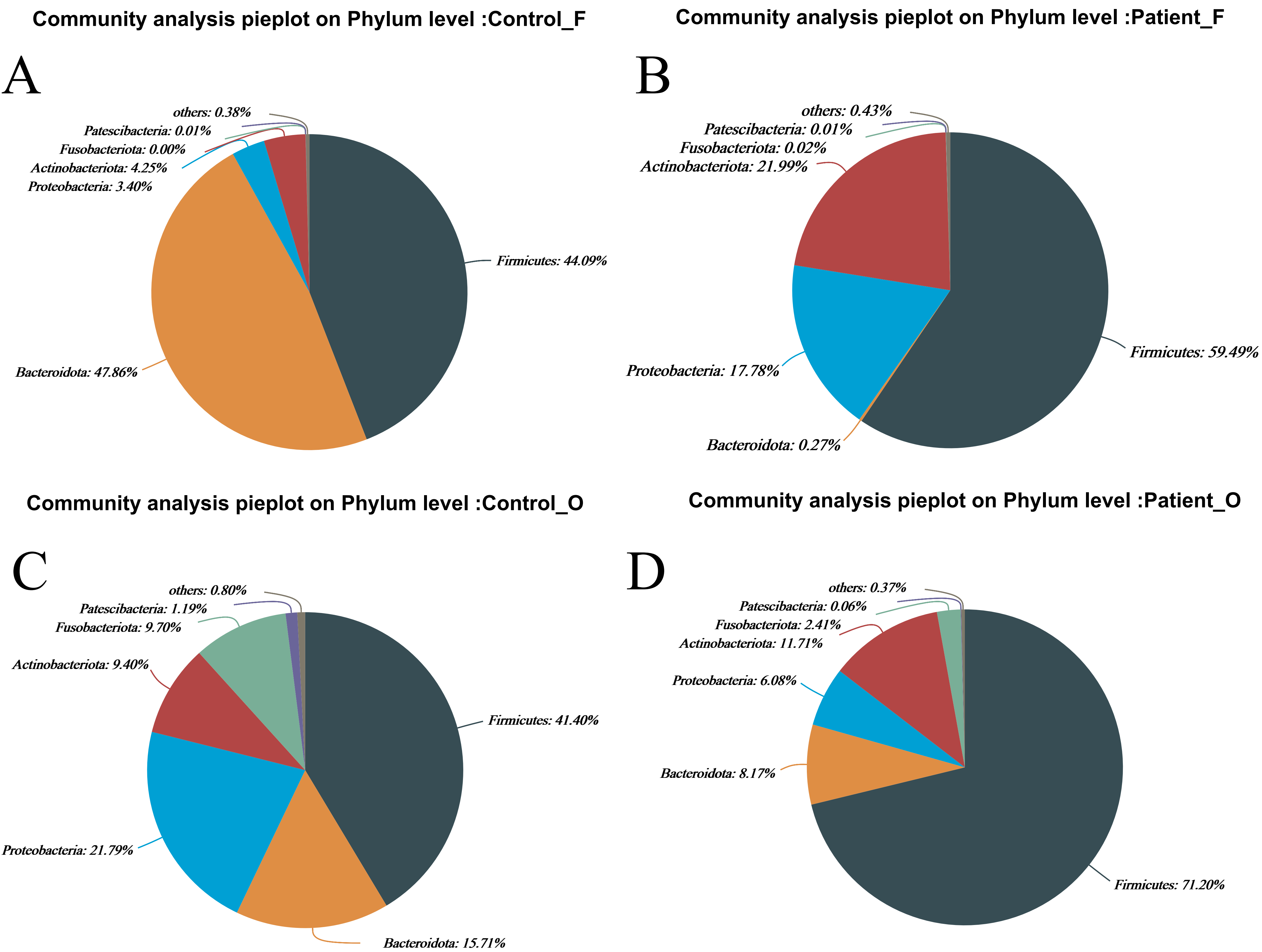
Community analysis pieplot on Phylum level :Control_F
A
others: 0.38%
Patescibacteria: 0.01%
Fusobacteriota: 0.00%
Actinobacteriota: 4.25%
Proteobacteria: 3.40%
Firmicutes: 44.09%
Bacteroidota: 47.86%
Community analysis pieplot on Phylum level :Patient_F
B
others: 0.43%
Patescibacteria: 0.01%
Fusobacteriota: 0.02%
Actinobacteriota: 21.99%
Firmicutes: 59.49%
Proteobacteria: 17.78%
Bacteroidota: 0.27%
Community analysis pieplot on Phylum level :Control_O
C
others: 0.80%
Patescibacteria: 1.19%
Fusobacteriota: 9.70%
Actinobacteriota: 9.40%
Firmicutes: 41.40%
Proteobacteria: 21.79%
Bacteroidota: 15.71%
Community analysis pieplot on Phylum level :Patient_O
D
others: 0.37%
Patescibacteria: 0.06%
Fusobacteriota: 2.41%
Actinobacteriota: 11.71%
Proteobacteria: 6.08%
Bacteroidota: 8.17%
Firmicutes: 71.20%
