# Supplementary material for: Alteration of the oral and gut microbiota in patients with Kawasaki disease
Source: PeerJ. 2023 Jul 10;11:e15662. doi: 10.7717/peerj.15662 (PMC10340105; doi:10.7717/peerj.15662)
Supplement: Supplemental Information 7 — Patient F and Control F represent fecal samples from KD patients and health, respectively; Patient O and Control O represent oral samples from KD patients and health, respectively [file peerj-11-15662-s007.pdf]

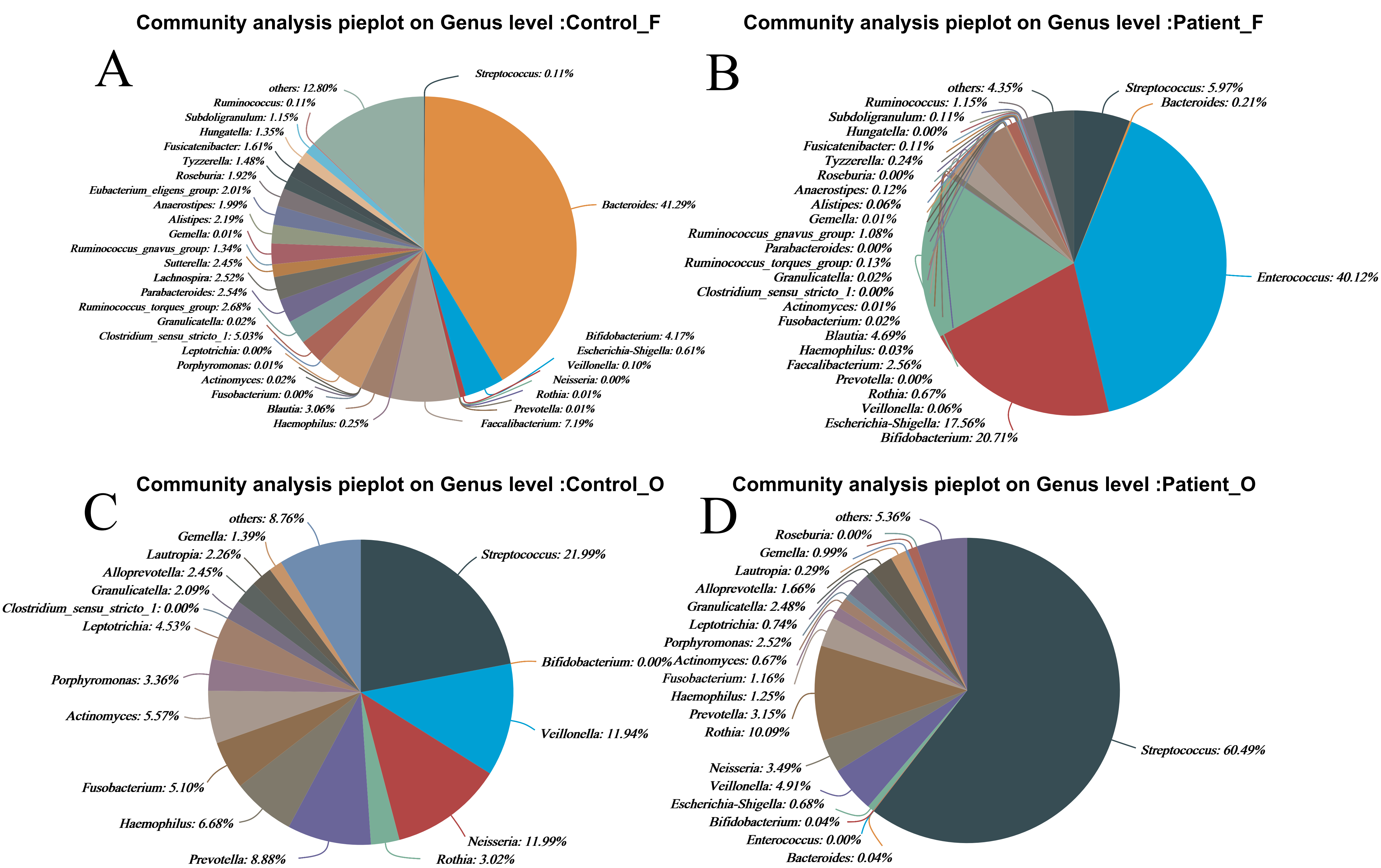
Community analysis pieplot on Genus level :Control_F
A
Streptococcus: 0.11%
others: 12.80%
Ruminococcus: 0.11%
Subdoligranulum: 1.15%
Hungatella: 1.35%
Fusicatenibacter: 1.61%
Tyzzerella: 1.48%
Roseburia: 1.92%
Eubacterium_eligens_group: 2.01%
Anaerostipes: 1.99%
Alistipes: 2.19%
Gemella: 0.01%
Ruminococcus_gnavus_group: 1.34%
Sutterella: 2.45%
Lachnospira: 2.52%
Parabacteroides: 2.54%
Ruminococcus_torques_group: 2.68%
Granulicatella: 0.02%
Clostridium_sensu_stricto_1: 5.03%
Leptotrichia: 0.00%
Porphyromonas: 0.01%
Actinomyces: 0.02%
Fusobacterium: 0.00%
Blautia: 3.06%
Haemophilus: 0.25%
Bacteroides: 41.29%
Bifidobacterium: 4.17%
Escherichia-Shigella: 0.61%
Veillonella: 0.10%
Neisseria: 0.00%
Rothia: 0.01%
Prevotella: 0.01%
Faecalibacterium: 7.19%
Community analysis pieplot on Genus level :Patient_F
B
others: 4.35%
Ruminococcus: 1.15%
Subdoligranulum: 0.11%
Hungatella: 0.00%
Fusicatenibacter: 0.11%
Tyzzerella: 0.24%
Roseburia: 0.00%
Anaerostipes: 0.12%
Alistipes: 0.06%
Gemella: 0.01%
Ruminococcus_gnavus_group: 1.08%
Parabacteroides: 0.00%
Ruminococcus_torques_group: 0.13%
Granulicatella: 0.02%
Clostridium_sensu_stricto_1: 0.00%
Actinomyces: 0.01%
Fusobacterium: 0.02%
Blautia: 4.69%
Haemophilus: 0.03%
Faecalibacterium: 2.56%
Prevotella: 0.00%
Rothia: 0.67%
Veillonella: 0.06%
Escherichia-Shigella: 17.56%
Bifidobacterium: 20.71%
Streptococcus: 5.97%
Bacteroides: 0.21%
Enterococcus: 40.12%
Community analysis pieplot on Genus level :Control_O
C
others: 8.76%
Gemella: 1.39%
Lautropia: 2.26%
Alloprevotella: 2.45%
Granulicatella: 2.09%
Clostridium_sensu_stricto_1: 0.00%
Leptotrichia: 4.53%
Porphyromonas: 3.36%
Actinomyces: 5.57%
Fusobacterium: 5.10%
Haemophilus: 6.68%
Prevotella: 8.88%
Rothia: 3.02%
Neisseria: 11.99%
Veillonella: 11.94%
Bifidobacterium: 0.00%
Streptococcus: 21.99%
Community analysis pieplot on Genus level :Patient_O
D
others: 5.36%
Roseburia: 0.00%
Gemella: 0.99%
Lautropia: 0.29%
Alloprevotella: 1.66%
Granulicatella: 2.48%
Leptotrichia: 0.74%
Porphyromonas: 2.52%
Actinomyces: 0.67%
Fusobacterium: 1.16%
Haemophilus: 1.25%
Prevotella: 3.15%
Rothia: 10.09%
Neisseria: 3.49%
Veillonella: 4.91%
Escherichia-Shigella: 0.68%
Bifidobacterium: 0.04%
Enterococcus: 0.00%
Bacteroides: 0.04%
Streptococcus: 60.49%
